# Effects of Executive Functions and Cognitive Variables in Experimentally Induced Acute Pain Perception during a Distraction Task: A Study on Asymptomatic Pain-Free Individuals

**DOI:** 10.3390/life14091141

**Published:** 2024-09-10

**Authors:** Angela Tejera-Alonso, Francisco G. Fernández-Palacios, Juan C. Pacho-Hernández, Arvin Naeimi, Ana I. de-la-Llave-Rincón, Silvia Ambite-Quesada, Ricardo Ortega-Santiago, César Fernández-de-las-Peñas, Margarita Cigarán-Mendez

**Affiliations:** 1 Department of Psychology, Universidad Rey Juan Carlos, 28922 Alcorcón, Spain; angela.tejera@urjc.es (A.T.-A.); juancarlos.pacho@urjc.es (J.C.P.-H.); arvin.naeimi@gmail.com (A.N.); margarita.cigaran@urjc.es (M.C.-M.); 2 Escuela Internacional de Doctorado, Universidad Rey Juan Carlos, 28922 Alcorcón, Spain; 3 Student Research Committee, School of Medicine, Guilan University of Medical Sciences, Rasht 41446-66949, Iran; 4 Department Physical Therapy, Occupational Therapy, Rehabilitation, and Physical Medicine, Universidad Rey Juan Carlos, 28922 Alcorcón, Spain; anaisabel.delallave@urjc.es (A.I.d.-l.-L.-R.); silvia.ambite.quesada@urjc.es (S.A.-Q.); ricardo.ortega@urjc.es (R.O.-S.); cesar.fernandez@urjc.es (C.F.-d.-l.-P.)

**Keywords:** distraction task, pain, cognition, attention, executive function

## Abstract

The aim of this study was to investigate the influence of executive functioning and cognitive performance on individual experimentally induced pain perception during distractor tasks in an asymptomatic pain-free population. A total of 59 healthy pain-free subjects (59.3% women, mean age: 46.5 ± 24.7 years) completed a battery test that assessed execution functions (cognitive flexibility, working memory, mental inhibition), attention level, and psychological aspects (anxiety/depressive levels—HADS, pain catastrophizing—PCS, pain anxiety symptoms—PASS 20, sleep quality—PSQI) before conducting two n-back distraction tasks. Pain was experimentally induced with a thermal stimulus that was able to induce moderate pain (70/100 points) and applied to the non-dominant forearm. The thermal stimulus was applied before and during both (one-back and two-back) distraction tasks. The analyses consisted of separated repeated-measures ANOVA that considered the functioning on each test (cognitive flexibility, working memory, mental inhibition, selective attention) and controlled for sociodemographic and psychological aspects by comparing the pain intensity at the baseline and during the one-back and two-back distractor tasks. All ANOVAs found a significant effect of the distraction task, which indicates that the perceived pain intensity scores were lower during the one-back and two-back tasks (*p* < 0.001) as compared with the baseline. No interaction effect between the distractor tasks and working memory (*p* = 0.546), mental inhibition (*p* = 0.16), cognitive flexibility (*p* = 0.069), or selective attention (*p* = 0.105) was identified. The current study found that a distraction task decreased the perceived intensity of experimentally induced pain in asymptomatic pain-free individuals and that this effect was not related to executive function or attention levels.

## 1. Introduction

Millions of patients annually suffer from pain because of trauma, illness, or surgery. Pain is the most common reason for admission to emergency departments, where it comprises more than 40% of the over 100 million visits in the United States of America each year [1]. Pain is a multidimensional personal experience that is affected by biological, psychological, and social factors [2]. Biopsychosocial pain models suggest that an individual’s thoughts, feelings, and behaviors can modulate pain perception [3]. This multidimensional nature accounts for inter-individual variability within the pain experience [2,4]. Experimental evidence strongly supports this multidimensional nature of the pain experience, thus indicating that pain perception involves a widely distributed neural network in pain processing [4,5]. Several cortical regions engaged in pain processing are also implicated in cognitive processes [6]. Therefore, it is not surprising that increasing evidence suggests a potential involvement of cognitive function in pain sensitivity. Besides the well-recognized role of the attentional control function in pain experience [7], studies underscore that other executive functions may also be crucial in this process [8].

Executive functions encompass advanced cognitive processes, such as sustained attention, response inhibition, working memory, and error processing, which enables subjects to direct their behavior toward goals in an adaptive manner [9]. Research indicates that in the realm of executive functioning, enhanced cognitive inhibition is linked to a reduction in sensitivity to pain and an increase in pain tolerance [10,11]. While these findings are promising, it remains unclear whether such associations are limited to cognitive inhibition domains or extend to other non-cognitive executive functions. Oosterman et al. [11] included several executive functions but found no correlation with experimental pain outcomes. However, their evaluation lacked a well-defined theoretical framework of executive functions, like the one proposed by Miyake et al. [9], which hindered the exploration of potential interactions between different executive functions. Miyake et al. [9] employed factor analyses on widely used neuropsychological and executive function tests and identified the following three factors that accounted for a significant portion of the variance: shifting between tasks or mental sets (shifting); updating or monitoring of working memory representations (updating); and suppressing dominant, automatic, or prepotent responses (inhibition). A comprehensive study into these three executive functions concerning experimentally induced pain in healthy populations is yet lacking.

Distraction is a psychological strategy commonly used for pain inhibition in individuals with chronic pain [12]. Based on the notion that focusing on stimuli unrelated to pain reduces the overall pain experience, distraction endeavors to divert attention away from the painful stimulus by engaging in a competing demand [1,13]. However, there is limited evidence regarding the effect of this strategy in the context of pain perception. The magnitude of the analgesic response of a distraction task varies across studies [14]. Although this disparity in findings may be attributed in part to differences in experimental paradigms and populations, evidence suggests that within-subject variation in executive functions, particularly cognitive inhibition abilities, could play a role in shaping pain perception [10,11,15] and may offer protection against pain-induced interference in task performance [14,16]. However, the impact of cognitive inhibition abilities on the efficacy of a distractive task remains unclear [14,16]. Additionally, the presence of mood disorders, such as anxiety or depression [17], as well as poor sleep [18], can also influence pain modulation. Nevertheless, scientific evidence is heterogeneous since the relevance of anxiety and depression is not consistent, and the effect of poor sleep is small but high for pain catastrophizing [17,18].

To the best of the author’s knowledge, there is a lack of scientific literature examining the connection between executive functioning and pain perception in adult populations. Consequently, due to the limited scientific evidence on this topic, this study investigated the potential influence of executive functioning and cognitive performance on experimentally induced pain perception after a distractor task. It was found that higher executive functioning is associated with a higher hypoalgesia of the distraction task [19], and thus, we hypothesized that subjects with higher executive and cognitive functioning would exhibit a higher pain reduction during the distraction tasks than those with lower executive and cognitive functioning. We used asymptomatic pain-free subjects to identify the influence of executive functions and cognitive performance in experimentally induced pain perception in a situation without the interference of chronic pain.

## 2. Methods

### 2.1. Study Design

This experimental study utilized a 2 × 3 mixed factorial design with two independent variables and two dependent variables. The first independent variable (within-subject factor) was the type of distracting task used (two levels: 1-back task and 2-back task), whereas the second independent variable (between-subject factor) was the level of functioning on each neurocognitive test (three levels: low, medium, high). The dependent variables were the perceived intensity of experimentally induced pain at the baseline and while performing the 1-back and 2-back distractor tasks.

### 2.2. Participants

Consecutive healthy adults aged between 20 and 65 years old were voluntarily recruited from local announcements on bulletin boards at Universidad Rey Juan Carlos, Madrid (Spain), and by social media platforms. A clinical examination was conducted to assess the exclusion criteria: (a) psychosis or significant psychiatric disorder; (b) taking tricyclic antidepressants (50 mg daily of amitriptyline or equivalent) or psychoactive medications (anticonvulsants, muscle relaxants, antipsychotics), except for low doses of benzodiazepines (10 mg daily of diazepam or equivalent); (c) a history of infectious diseases, metabolic diseases, renal diseases, endocrine diseases, neuromuscular diseases, oncological diseases, or chronic pain; (d) a history of surgery within the last 10 years; (e) pregnancy or lactation; (f) mental disability; (g) cognitive and sensory disorders; (h) caffeine intake in the 2 h prior to measurement; (i) intense physical activity on the day of the test; (j) lack of consent to participate in this study; and (k) having a score under 27 in the Mini-Mental State Examination (MMSE) screening test [20]. The MMSE is a short screening test used to measure cognitive functioning. It evaluates the areas of orientation, attention and calculation, memory, and language. Scores equal to or above 27/30 points are considered normal.

The study protocol received approval from the Ethics Committee of Universidad Rey Juan Carlos (URJC 1909202332123) in accordance with the Declaration of Helsinki. All participants signed the written informed consent prior to their inclusion in this study.

### 2.3. Sample Size Calculation

We calculated the sample size with G*Power software v. 3.1.9.2. (Dusseldorf, Germany) with the following input parameters: α = 0.05, a target power of 0.80, and a medium effect size (f^2^ = 0.25). An effect size of small-to-moderate magnitude (0.25) was set according to Cohen’s criteria [21] to detect clinically significant differences. Given the cross-sectional nature of this study, no participant dropouts were anticipated. Therefore, a sample size of at least 42 participants was determined.

### 2.4. Psychological Variables

The Hospital Anxiety and Depression Scale (HADS) was employed to evaluate anxiety and depressive symptoms [22]. The HADS consists of 14 items with a Likert-type response format of 4 points ranging from 0 to 3 points. The adapted Spanish version by Vallejo et al. [23] shows, like the original version, a 2-factor solution: anxiety symptoms (HADS-A, 7 items, 0–21 points) and depressive symptoms (HADS-D, 7 items, 0–21 points).

The Spanish version of the Pain Catastrophizing Scale (PCS) was used to measure the degree of catastrophic thinking in the presence of anticipated pain. The PCS consists of 13 items with a 5-point Likert response ranging from 0 (Not at all) to 4 (All the time) [24]. The Spanish-validated version presents a three-factor solution, like the original version: magnification (3 items), rumination (4 items), and helplessness (6 items) [25].

The short version of the Pain Anxiety Symptoms Scale-20 (PASS-20) was used to evaluate pain anxiety [26]. The PASS-20 is a 20-item scale that assesses cognitive anxiety (“I worry when I have pain”), avoidance and escape behaviors (“I try to avoid activities that cause pain”), fear of pain (“pain sensations are terrifying”), and physiological anxiety (“pain makes me nauseous”) using a 6-point Likert scale from 0 (never) to 5 (always) [26].

The Pittsburgh Sleep Quality Index (PSQI), which consists of 19 items that provide an overall score (0–21 points), was used to evaluate the quality of sleep [27]. The PSQI has shown adequate reliability, construct validity, and discriminant validity [27].

### 2.5. Executive Functions

Different authors studied the influence of various cognitive functions on pain perception. Among the executive functions, working memory, mental inhibition, and cognitive flexibility showed the highest relevance for these processes [28,29,30,31].

The “D/R/I Digits” subtest of the Wechsler Adult Intelligence Scale battery (WAIS-IV) was used to evaluate immediate memory and working memory [32]. This subtest uses sequencing skills, planning, alertness, and cognitive flexibility. It is composed of three tasks: Forward Digit Span (repeating a series of digits in the same order as presented orally), Backward Digit Span (repeating a series of digits in reverse order), and Sequencing Digit Span (repeating a sequence of numbers presented by the examiner in order from the smallest to the highest).

The “response inhibition index” of the Five-Digit Test (FDT), which is a STROOP-like task, was used to assess mental inhibition and cognitive flexibility [33]. The FDT comprises four components—reading, counting, choice, and alternation—each with varying levels of difficulty for assessing executive functions and they are applied sequentially. Each component of the test contains 50 items. The reading and counting components assess automatic and simple processes, whereas the alternation and choice components evaluate more complex processes, as they require active mental control and demand voluntary effort, which slows down the response speed. The resulting scores are as follows: Decoding_FDT, duration in seconds needed to read all numeric elements; Retrieving_FDT, duration in seconds needed to read all non-numeric elements (e.g., asterisks); Inhibiting_FDT, duration in seconds to read the group of the same numeric item; and Shifting_FDT, time in seconds to read the group of the same numeric item mixed with numeric items within a box. Finally, two complementary scores can be obtained: one for response inhibition and one for mental flexibility [33].

### 2.6. Attention Assessment

Attention is also a relevant cognitive process in the pain experience since it diverts the person’s attentional resources toward the stimulus that is causing pain [34,35,36,37]. Accordingly, the Spanish version of the D2 Attention Test, which assesses mental concentration, was employed [38,39]. This time-limited assessment aims to gauge the ability to focus on relevant aspects of a task while ignoring irrelevant ones, alongside the capacity to complete the task swiftly, continuously, and accurately. It comprises 14 lines, each containing 47 characters, totaling 658 elements. The lines feature the letters ‘d’ and ‘p’, sometimes accompanied by one or two small dashes positioned above or below the letters.

Participants must meticulously scan each line from left to right, marking every ‘d’ with two small dashes (above, below, or both). These are considered relevant elements, while other combinations such as ‘p’ with or without dashes and ‘d’ with either one dash or none are deemed irrelevant. Participants have 20 s per line. The scores obtained include TR (total responses), TA (correct responses, which represent correct relevant elements), O (omissions, which are relevant elements that were not marked), C (commissions, which are irrelevant elements that were marked), TOT (total test effectiveness, calculated as TR minus the sum of O and C), CON (concentration index, which is determined as TA minus C), TR+ (the line with the highest number of tested elements), TR− (the line with the lowest number of tested elements), and VAR (variation index, which is calculated as TR+ minus TR−). In the current study, only the main measure (TOT) of the D2 test was used.

### 2.7. Distraction Task

Distraction tasks are one of the most used strategies to reduce pain perception, which involves diverting attention from the painful stimulus by refocusing attention on another task [30,36,40].

An n-back task was used as a distraction. The n-back tasks consist of a series of letters or numbers that appear in a row. The participant performing the n-back task must determine whether the stimuli showing are the same or not as the one they saw previously, depending on the n-back number (i.e., 1-back, 2-back, 3-back, etc.). Both 1-back and 2-back were programmed using E-prime 3.0 software, which comprised 10 series with 21 letters each. In the 1-back task, participants indicated whether each letter matched the previous one by pressing ‘1’ for ‘yes’ and ‘2’ for ‘no’. In the 2-back task, they determined whether each letter matched the one from two trials ago. The presentation time for each letter was 840 ms, preceded by a 1000 ms fixation cross [30]. Each series contained 30% repeating letters, with no more than three consecutive repetitions. The series duration-matched that of the painful stimulation for synchronized timing.

### 2.8. Experimental Study Procedure

Subjects that met the inclusion criteria were assessed at the experimental clinical psychology laboratory of Universidad Rey Juan Carlos between October 2023 and March 2024. They fulfilled all the tests about working memory, mental inhibition, cognitive flexibility, and selective attention, which lasted approximately 20 min (Table 1). The study protocol for each individual session lasted 90 min and was conducted by an experienced clinical neuropsychologist.

First, a pain calibration task that aimed to experimentally induce moderate pain in each subject was conducted using a thermal stimulus. Moderate pain was defined as a score of 70 out of 100 on a computerized visual analog pain scale (CoVAS) from 0 to 100 mm [41,42]. The thermal stimulus was applied with a Thermotest System (Somedic AB^®^, Sweden). A 30 × 30 mm thermode was positioned on the non-dominant forearm, 15 cm below the wrist [38]. Participants received heat thermal stimuli using a “ramp and hold” procedure. Each stimulus began at 32 °C for 3 s; it was increased by 0.7 °C until the temperature-induced moderate pain (70/100 on the CoVAS). They were instructed to press the stop button when that level of pain (70/100 on the CoVAS) was induced. Afterward, the temperature decreased progressively until returning to 32 °C. A 30 s rest period between stimuli was used. The pain calibration task ended when three consecutive pain intensity scores were ≥70/100 mm on the CoVAS. The thermal stimulus was calculated as the arithmetic mean of the three trials. In the current study, the mean temperature of the thermal stimulus needed for inducing moderate pain in the total sample was 46.7 (SD: 1.2) °C.

Second, participants learned to perform distraction tasks, including the 1-back and 2-back tasks, by engaging complex executive functions, like working memory [37]. They completed a brief three-series training, which involved receiving visual feedback and aiming for at least 80% accuracy. Afterward, the participants underwent two sets of ten painful stimuli each, identical to those used in the pain calibration task and lasting approximately 30 s each. While receiving the first set of stimuli, participants completed 10 trials of the 1-back task, and during the second set, they also completed 10 trials of the 2-back task. After each pain stimulus, the participants rated their perceived pain intensity using the CoVAS scale from 0 to 100. The pain intensity during each distraction task was calculated as the mean of the provided intensities during the distraction task. The order of application of the distraction tasks was randomized to avoid sequential or accumulative effects.

### 2.9. Statistical Analysis

SPSS version 27 Statistical Software was used to conduct the statistical analyses, where the results were considered significant at the level *p* < 0.05. As in a prior study [43], the presence of outliers was analyzed using boxplots: those values below the first quartile minus 1.5 × the interquartile range and those values above the third quartile plus 1.5 × the interquartile range were considered as potential outliers (no outliers were identified in the current study). The normality of the data was tested using the Kolmogorov–Smirnov test (only data from the dependent variables and D2_TOT, response inhibition, mental flexibility, and working memory had a normal distribution).

Subsequently, descriptive and frequency analyses were undertaken for the sociodemographic, psychological, and cognitive variables. First, Spearman’s correlation analyses were conducted for the sociodemographic, psychological, neurocognitive, and dependent variables with the aim of determining potential covariates to include in the subsequent analyses. Significant correlations between the dependent variables and age, marital status, employment status, and pain anxiety were found. Therefore, these variables were incorporated as covariates in the subsequent analyses.

Second, to analyze the main hypothesis of this study, four repeated-measures analyses of variance (ANOVA) were performed to assess the effect of functioning on each neurocognitive test (low, medium, and high levels) on the perceived pain intensity at the baseline and during the 1-back and 2-back distractor tasks. Hence, the perceived pain intensity (baseline, 1-back, and 2-back) was the within-subject factor, and the level of functioning on each neurocognitive test was the independent variable. Age, marital status, employment status, and pain anxiety were included as covariates. The D2_TOT (selective attention, D2 test), mental inhibition and mental flexibility (Five Digit Test), and working memory (D/R/I Digits) indices were used in the main analyses. In accordance with the interpretation rules of the test manual for each instrument, the centile score of each of these indices was calculated, and this score was subsequently classified into low, medium, and high levels. Prior to the execution of the test, the assumptions of normality and sphericity were examined and they were met. The effect sizes were determined using the partial eta squared (n^2^_p_). Cohen [21] defines a small effect as 0.01, a medium effect as 0.06, and a large effect for values greater than 0.14. To identify specific intergroup differences, Bonferroni post hoc tests were performed.

## 3. Results

### 3.1. Descriptive Data

The study sample was composed of 59 healthy pain-free subjects (59.3% women, mean age: 46.5, SD: 24.7 years). The sociodemographic, psychological, and neurocognitive data of the sample can be observed in Table 2.

### 3.2. Preliminary Analysis: Spearman’s Correlation Analyses

The Spearman’s correlation analyses revealed that the perceived pain intensity during the one-back task was negatively associated with age (r = −0.263, *p* = 0.044), marital status (r = −0.308, *p* = 0.018), and employment status (r = −0.289, *p* = 0.026), and positively related to pain anxiety (r = 0.335, *p* = 0.010). Furthermore, the perceived pain intensity during the two-back task was also negatively associated with age (r = −0.281, *p* = 0.031), marital status (r = −0.388, *p* = 0.002), and employment status (r = −0.353, *p* = 0.006), and positively associated with pain anxiety (r = 0.352, *p* = 0.006).

### 3.3. Effect of Working Memory on Pain Analgesia

The repeated-measures ANOVA that analyzed the effect of working memory on perceived pain intensity during the one-back and two-back tasks (estimated marginal means and standard deviations are shown in Table 3) found a main effect of the distractor task (Wilk’s λ = 0.432, F [2, 51] = 33.556, *p* < 0.001, n^2^_p_ = 0.568, β-1 = 0.999). Post hoc analyses indicated that the perceived pain intensity scores were lower during the one-back task (mean difference: 26.0, SD: 3.8, 95% CI 16.6 to 35.4, *p* < 0.001) and in the two-back task (mean difference: 29.5, SD: 3.5, 95% CI 20.6 to 38.3, *p* < 0.001) as compared with the baseline. No significant differences in the perceived pain intensity scores between the one-back and two-back tasks (mean difference: 3.4, SD: 2.0, 95% CI −1.5 to 8.5, *p* = 0.283) were found. Additionally, no significant interaction effect between the distractor task and the level of functioning in working memory (Wilk’s λ = 0.942, F [4, 102] = 0.772, *p* = 0.546, n^2^_p_ = 0.029, β-1 = 0.240) was found after controlling for age, marital status, employment status, and pain anxiety.

### 3.4. Effects of Mental Inhibition on Pain Analgesia

The repeated-measures ANOVA that analyzed the effect of mental inhibition on the perceived pain intensity during the one-back and two-back tasks (Table 3) showed a significant main effect of the distractor task (Wilk’s λ = 0.511, F [2, 51] = 24.432, *p* < 0.001, n^2^_p_ = 0.489, β-1 = 0.999). Pairwise post hoc analyses indicated that the perceived pain intensity scores were lower in both the one-back (mean difference: 33.5, SD: 5.8, 95% CI 19.06 to 47.8, *p* < 0.001) and two-back (mean difference: 37.7, SD: 5.3, 95% CI 24.5 to 51.0, *p* < 0.001) tasks as compared with the baseline. No significant difference in the perceived pain intensity scores between the one-back and two-back tasks (mean difference: 4.2, SD: 3.2, 95% CI ranging from −3.8 to 12.2, and a *p* = 0.594) was found. Furthermore, no significant interaction effect between the distractor task and the level of functioning in mental inhibition (Wilk’s λ = 0.880, F [4, 102] = 1.683, *p* = 0.160, n^2^_p_ = 0.062, β-1 = 0.501) was seen after controlling for age, marital status, employment status, and pain anxiety.

### 3.5. Effects of Cognitive Flexibility on Pain Analgesia

The estimated marginal means and standard deviations reflecting the effect of cognitive flexibility on perceived pain intensity during the one-back and two-back tasks are presented in Table 3. The results show a main effect of the distractor task (Wilk’s λ = 0.500, F [2, 51] = 25.459, *p* = 0.001, n^2^_p_ = 0.500, β-1 = 0.999). Post hoc analyses indicated that the perceived pain intensity scores were lower in the one-back task (mean difference: 27.2, SD: 5.6, 95% CI 13.2 to 41.2, *p* < 0.001) and in the two-back task (mean difference: 34.4, SD: 4.9, 95% CI 22.1 to 46.7, *p* < 0.001) as compared with the baseline. No significant difference in the perceived pain intensity scores was found (mean difference: 7.2, SD: 2.9, 95% CI ranging from −0.1 to 14.5, and a *p* = 0.058) between the one-back and two-back tasks. The data did not reveal a significant interaction effect between the distractor task and the level of functioning in cognitive flexibility (Wilk’s λ = 0.809, F [4, 102] = 2.845, *p* = 0.069, n^2^_p_ = 0.074, β-1 = 0.756) after controlling for age, marital status, employment status, and pain anxiety.

### 3.6. Effects of Attention Levels on Pain Analgesia

Estimated marginal means and standard deviations reflecting the effect of selective attention on perceived pain intensity during the one-back and two-back tasks are shown in Table 3. The ANOVA showed a significant main effect of the distractor task (Wilk’s λ = 0.422, F [2, 51] = 34.875, *p* < 0.001, n^2^_p_ = 0.578, β-1 = 0.999). The pairwise post hoc analyses indicated that the perceived pain intensity scores were lower both in the one-back task (mean difference: 28.6, SD: 4.3, 95% CI 17.9 to 39.3, *p* < 0.001) and in the two-back task (mean difference: 33.1, SD: 3.9, 95% CI 23.3 to 42.8, *p* < 0.001) as compared with the baseline. No significant difference in the perceived pain intensity scores was found between the one-back and two-back tasks (mean difference: 4.4, SD: 2.3, 95% CI −1.2 to 10.2, *p* = 0.177). No significant interaction effect between the distractor task and the level of functioning in attention level (Wilk’s λ = 0.862, F [4, 102] = 1.965, *p* = 0.105, n^2^_p_ = 0.072, β-1 = 0.573) after controlling for age, marital status, employment status, and pain anxiety was observed.

## 4. Discussion

This study found that a distraction task decreased the perceived intensity of experimentally induced pain and that this effect was not related to executive functioning, attention levels, and psychological variables in a sample of asymptomatic pain-free individuals. These results did not support the initial hypothesis of this study since a higher level of executive functioning was not associated with a more analgesic effect of a cognitive distraction task.

### 4.1. Induced Pain Analgesia and Distraction Task

Distraction tasks are cognitive strategies used to reduce pain based on the premise that diverting attention from the pain source can reduce the pain experience; however, their effect seems to depend on the modality (e.g., visual, tactile, auditive) of the distraction [36]. In the current study, we investigated the effect of visual distraction tasks (n-back) on the experimentally induced acute pain. So far, some studies investigated the impact of various visual distractor tests on acute [35,44,45,46,47] and chronic [48] pain. Consistent with our findings, numerous authors exhibited that visual distraction is related to a lower level of reported acute pain [35,44,45,46]. For instance, Bantick et al. [35] examined the effects of cognitive demand on pain perception using a modified Stroop task, which is known as the Stroop counting task, with two levels of difficulty (high and low; counterbalanced). In this study, eight pain-free healthy adults performed this task while experiencing experimental acute pain induced by a heat pulse stimulus and showed that the participants reported a lower pain intensity when engaged in the higher cognitive demand version of the task [35]. However, Stancak et al. found different results when comparing two counterbalanced distraction conditions [47]. In their study, 24 pain-free healthy subjects were instructed to count the figures in the Rubin vase optical illusion (distraction task) while experiencing experimentally induced acute pain from laser pulses on one hand; while in the focusing condition, they were asked to concentrate on the painful sensation. The authors found that the intensity of the reported pain did not differ between both conditions, which contrasted with current the findings [47].

Overall, distraction mechanisms for acute pain can be understood through cognitive, learning, and neurobiological perspectives [49]. Cognitive theories, such as the limited attentional capacity and multiple resource theories, suggest that distraction tasks reduce the attention to pain by utilizing attentional resources, with more effective distractions competing for the same resources used to process pain [50,51]. In this regard, the neurocognitive model indicates that distraction tasks work by modulating involuntary pain attention through a voluntary focus on specific stimuli, though it is less effective when the pain is highly salient, unless goal-directed motivation is present [49,52]. Moreover, learning processes based on behavioral theory propose that distraction prevents the formation of a conditioned fear response to pain by diverting the focus away from painful stimuli and fostering relaxation [50]. On the other hand, neurobiological evidence from neuroimaging shows that distraction alters brain activation by decreasing activity in pain-processing areas, like the thalamus and somatosensory cortices, and by increasing it in areas associated with pain modulation, such as the periaqueductal gray and cingula and frontal cortices [49,53,54]. A more recent study identified that brain responses during pain analgesia, i.e., enhanced activity at the level of the dorsolateral prefrontal cortex and the posterior parietal cortex, is the same independent of the nature of the distraction task [55].

It is important to note that the magnitude of the analgesic response of distraction tasks also varies across published studies [14], where some authors have reported significant effects [37,40], others have reported small effects [56], and others have reported no effect at all [57]. It seems that the effectiveness of different types of distractions (e.g., cognitive, visual, auditory); individual differences in cognitive abilities; the type, intensity, and duration of pain; the study environment; and smaller sample sizes could all potentially explain these discrepancies.

In fact, the magnitude of the effect of the distraction task on pain analgesia can be influenced by several factors. Probably one of the most important factors is the population investigated. Thus, most published studies included clinical populations with acute or chronic pain conditions [36]. Evidence supports the presence of sensitization mechanisms, particularly in chronic pain conditions [58]. Accordingly, it would be expected that the analgesic response of a distraction task would be smaller in individuals with central sensitization. Similarly, albeit to a lesser extent than in chronic pain, acute pain also features signs of sensitization. Hence, the use of acute pain patients may also lead to a smaller effect on pain modulation with a distraction task. In the current study, we used symptomatic pain-free individuals to avoid the presence of signs of sensitization to the analgesic effect of a distraction task on experimentally induced pain.

The analgesic effect of distraction tasks has mainly been attributed to central mechanisms related to a modulation of the activity of sensory and affective brain areas, e.g., by decreasing the activity of the thalamus, insula, primary and secondary somatosensory cortexes, or anterior cingulate cortex, and by increasing the activity of other areas, including the periaqueductal grey substance [54,59]. In such a scenario, according to the neurocognitive model of attention [52], the distraction task would be considered a top-down strategy that is able to decrease the attention on the painful stimuli (bottom-up input). Thus, all top-down processes can be affected, but the extent of attentional effort needed and what is accordingly regarded as goal-relevant information could be affected by other motivational factors, e.g., executive functions or attention levels (see next heading of this discussion).

The role of cognitive function in the analgesic response of a distraction task is supported by the fact that distraction tasks that need higher cognitive demand induce greater pain analgesia than distraction tasks that need less cognitive demand [31]. However, it is important to consider that cognitive fatigue has been recently shown to be able to impair performance on subsequent distraction tasks, which could decrease the individual’s ability to perform the task and to reduce their pain perception [60]. We did not find differences in the analgesia induced by both distraction tasks used in the current study, albeit the two-back task used has a higher cognitive demand than the one-back task. Two potential explanations could explain these results: (1) the execution of both distraction tasks was random between participants, which may have decreased the cognitive fatigue of the subjects, or (2) the difference in cognitive demand between both distractions tasks was not enough to stimulate pain analgesia to a greater extent.

### 4.2. Distraction Task, Pain Analgesia, and Cognitive Behaviors

It was hypothesized that the effect on pain modulation of distraction tasks could be lower if the individual exhibits high levels of pain catastrophizing or pain-related threat [53,61]. Our results did not observe an effect of either cognitive behaviors (pain catastrophizing, sleep quality) or psychological/emotional (anxiety, depression) aspects in the analgesic effect induced by the distraction tasks used in the current study. The use of a pain-free population, which, as expected, showed low anxiety, depression, and pain-catastrophizing levels, as well as normal sleep quality, could explain the current results. A recent study has observed that the role of pain catastrophizing on pain analgesia depends on the type of distraction task. In this study, pain catastrophizing had a greater role in pain analgesia with a social rather than with a sensory distraction [62]. Nevertheless, this study did not control for other psychological variables, such as anxiety or depressive levels, which could also interact with pain catastrophizing.

To date, conflicting data exist regarding the perception of experimentally induced pain in depressed patients. Some studies indicate that depressed patients exhibit lower pain thresholds [63,64,65], while others suggest higher pain thresholds [66,67,68,69]. In this regard, a meta-analysis of 32 studies (*n* = 1317) found no significant differences in pain intensity ratings, pain affect, and pain tolerance between individuals with and without depression [70]. Thus, conflicting results also exist regarding anxiety. While some studies have reported that anxiety predicts pain intensity [71,72], others found that anxiety is not a significant predictor [14]. Furthermore, in line with our findings, some studies indicated no correlation between pain catastrophizing and executive function performance [73]. It is possible that maladaptive coping strategies are more related to pain beliefs than to experimentally induced pain perception [73,74]. Nevertheless, it is possible that this lack of effect could be identified with other psychological variables not measured in this study.

### 4.3. Distraction Task, Pain Analgesia, and Executive Functions

Given that executive function and cognitive abilities, particularly those related to inhibition, can play a role in the pain experience [10,11,15], we aimed to identify the effects of executive functions that were shown to have the highest relevance for a pain experience, such as working memory, mental inhibition, and cognitive flexibility [28,29,30,31], on the analgesic effect of a distraction task. We did not find such an association in a sample of asymptomatic pain-free subjects. In other words, our findings did not observe any significant correlation between various aspects of executive function (cognitive flexibility, mental inhibition, working memory, and selective attention) and experimentally induced pain. Consistent with our results, Verhoeven et al. found that individuals with better executive functioning abilities (inhibition, task switching, and working memory) did not experience greater pain reduction during a distraction task than those with lesser abilities [14]. However, limited studies that investigated the correlation between executive functions and perception in acute pain settings were undertaken. Furthermore, although several studies focused on clinical pain experience and cognition in chronic pain patients, the results were inconsistent, with negative [75,76] and positive [77,78] associations observed between the pain experience and executive functions. In this regard, some studies revealed inconsistent findings between different aspects of executive function and perceived pain. Elkana et al. demonstrated that perceived pain is associated with two aspects of executive functions, such as inhibition and set shifting, but not with updating [73]. In contrast, others found that lower performance on a working memory task is associated with greater intense pain perception [79]. A systematic review indicated that while executive functioning may negatively correlate with responsiveness to experimental pain, this correlation is weak (very small effect sizes) and only 20% of the studies found a significant correlation [80]. Overall, the discrepancies may have been due to various factors, including the tests selected for evaluating various aspects of executive functions, the variety of acute/chronic pain conditions, different methods for inducing pain (such as heat or cold stimulus), and the diverse populations included. Further research is needed to evaluate the role of executive function on pain perception in different pain settings to confirm or refute our findings.

### 4.4. Limitations of This Study

The results of the current study should be considered within the study design and its potential limitations. First, we included pain-free asymptomatic subjects; accordingly, the current data cannot be extrapolated to clinical populations with pain, where the interaction of pain with the variables assessed in our study is more complex. Second, the study design was based on an experimentally induced acute pain model, and thus, the analgesic effect induced by the distraction tasks should not be applied to a chronic pain setting. Third, we only used a visual distraction task, and thus, we do not currently know the effect of neurocognitive variables and executive functions in pain analgesia induced by other types of tasks. Fourth, although the total sample was calculated for detecting moderate effects, the sample size was small for the comparison of some subgroups; hence, statistical analyses could have included type II errors. Future studies using the same study design and including clinical populations with different pain conditions are needed to confirm or refute the direction of the current findings.

## 5. Conclusions

The current study revealed that a distraction task (one-back and two-back) reduced the perceived intensity of experimentally induced pain in asymptomatic, pain-free individuals. This effect was neither associated with various aspects of executive function, such as working memory, mental inhibition, cognitive flexibility, and selective attention, nor with attention levels. Future studies in clinical pain populations with chronic pain conditions should be conducted to determine the analgesic response with the distraction tasks used in the current study in a clinical setting.

## Figures and Tables

**Table 1:** Cognitive domains and neuropsychological tasks.

Cognitive Domains	Neuropsychological Tests	Outcomes	Method of Administration
Cognitive screening	MMSE	Cognitive status	Auditory/visual/manual (paper)
Working memory	Digit Span Forward (WAIS-IV)	Span of digits	Auditory/oral
	Digit Span Backward (WAIS-IV)	Auditory working memory	
	Digit Span Sequencing (WAIS-IV)	Auditory working memory	
Selective attention	D2 Test of Attention	D2_TR	Visual/manual (paper)
		D2_TA	
		D2_TOT	
		D2_CON	
		D2_VAR	
		D2_O	
		D2_C	
Mental inhibition and cognitive flexibility	Five Digits Test FDT	Inhibiting_FDT	Visual/oral
		Shifting_FDT	
		Decoding_FDT	
		Retrieving_FDT	

MMSE: Mini-Mental Scale Examination; WAIS-IV: Wechsler Intelligence Scale for Adults-IV; D2_TR: total number of items answered; D2_TA: number of items answered correctly; D2_O: errors of omission committed; D2_C: commission errors made; D2_TOT: number of elements processed minus the total number of errors committed; D2_CON: number of relevant elements marked minus the number of commissions; D2_VAR: variation index; DSF: Digit Span Forward; DSB: Digit Span Backward; DSS: Digit Span Sequencing; Decoding_FDT: time in seconds to read all numeric items; Retrieving_FDT: time in seconds to read all non-numeric items; Inhibiting_FDT: time in seconds to read numeric items; Shifting_FDT: time in seconds to read non-numeric items.

**Table 2:** Descriptive statistics of the sociodemographic, psychological, and neurocognitive data.

Data	Mean (Standard Deviation)
Age (mean, SD)	46.4 (24.7)
Gender (*n*, %)	
Male	24 (40.7%)
Female	35 (59.3%)
Marital status (*n*, %)	
Single	26 (44.1%)
Married	25 (42.4%)
Divorced	4 (6.8%)
Widowed	4 (6.8%)
Educational level (*n*, %)	
Primary school	1 (1.7%)
Middle school	44 (74.6%)
High school	14 (23.7%)
Employment status (*n*, %)	
Student	25 (42.4%)
Working	5 (8.5%)
Unemployed	1 (1.7%)
Retired	28 (47.5%)
Psychological data	
Anxiety (HADS-A, 0–21; mean, SD)	6.5 (3.2)
Depression (HADS-D, 0–21; mean, SD)	3.1 (2.4)
Pain catastrophizing (PCS, 0–52; mean, SD)	15.8 (9.0)
Pain anxiety (PASS-20, 0–100; mean, SD)	27. 2 (12.0)
Sleep quality (PSQI, 0–21; mean, SD)	7.4 (3.4)
Neurocognitive data	
Mental inhibition (Inhibiting_FDT; mean, SD)	17.1 (7.1)
Cognitive flexibility (Shifting_FDT; mean, SD)	28.2 (10.9)
Selective attention (D2_TOT; mean, SD)	392.3 (91.4)
Working memory (D/R/I Digits; mean, SD)	23.9 (5.5)

*n*: the number of subjects; SD: standard deviation; HADS-A: anxiety dimension of the Hospital Anxiety and Depression Scale; HADS-D: depression dimension of the Hospital Anxiety and Depression Scale; PCS: Pain Catastrophizing Scale; PASS-20: Pain Anxiety Symptoms Scale-20; PSQI: The Pittsburgh Sleep Quality Index; FDT: Five Digits Test; D2: D2 Test of Attention; D/R/I Digits: Working memory subtest of the Wechsler Adult Intelligence Scale battery.

**Table 3:** Estimated marginal means and standard deviations of the repeated-measures ANOVA for each neurocognitive domain.

Working Memory							
	Low (*n* = 14)	Medium (*n* = 32)	High (*n* = 13)	Univariate tests			
	Mean (SD)	Mean (SD)	Mean (SD)	F	*p*	n^2^_p_	β-1
Baseline pain intensity	70.0 (0.0)	70.0 (0.0)	70.0 (0.0)			1.0	
Pain intensity 1-back task	40.6 (7.5)	50.7 (4.7)	40.5 (7.9)	0.989	0.379	0.037	0.213
Pain intensity 2-back task	36.1 (7.0)	44.2 (4.5)	41.0 (7.4)	0.476	0.624	0.018	0.124
Attention							
	Low (*n* = 9)	Medium (*n* = 40)	High (*n* = 10)	Univariate tests			
	Mean (SD)	Mean (SD)	Mean (SD)	F	*p*	n^2^_p_	β-1
Baseline pain intensity	70.0 (0.0)	70.0 (0.0)	70.0 (0.0)			1.0	
Pain intensity 1-back task	34.5 (8.9)	50.4 (4.2)	39.1 (8.5)	1.633	0.205	0.059	0.330
Pain intensity 2-back task	24.3 (8.1)	45.8 (3.8)	40.4 (7.7)	2.772	0.072	0.096	0.523
Mental Inhibition							
	Low (*n* = 12)	Medium (*n* = 44)	High (*n* = 3)	Univariate tests			
	Mean (SD)	Mean (SD)	Mean (SD)	F	*p*	n^2^_p_	β-1
Baseline pain intensity	70.0 (0.0)	70.0 (0.0)	70.0 (0.0)			1.0	
Pain intensity 1-back task	35.9 (8.6)	50. 4 (4.0)	23.0 (15.2)	2.334	0.107	0.082	0.452
Pain intensity 2-back task	28.0 (7.8)	46.6 (3.7)	21.9 (13.9)	3.226	0.048	0.110	0.591
Cognitive Flexibility							
	Low (*n* = 8)	Medium (*n* = 47)	High (*n* = 4)	Univariate tests			
	Mean (SD)	Mean (SD)	Mean (SD)	F	*p*	n^2^_p_	β-1
Baseline pain intensity	70.0 (0.0)	70.0 (0.0)	70.0 (0.0)			1.0	
Pain intensity 1-back task	31.2 (10.4)	48.3 (3.9)	48.7 (14.2)	1.158	0.322	0.043	0.243
Pain intensity 2-back task	16.4 (9.1)	45.6 (3.4)	44.5 (12.5)	4.352	0.018	0.143	0.730

## Data Availability

Materials and analysis code for this study are not available in any repository; however, we will make our data accessible upon request to the corresponding author.
